# Risk Factors for Intensive Care Unit Admission in Patients with Autoimmune Encephalitis

**DOI:** 10.3389/fimmu.2017.00835

**Published:** 2017-07-28

**Authors:** Gayane Harutyunyan, Larissa Hauer, Martin W. Dünser, Tobias Moser, Slaven Pikija, Markus Leitinger, Helmut F. Novak, Wolfgang Aichhorn, Eugen Trinka, Johann Sellner

**Affiliations:** ^1^Department of Neurology, Christian Doppler Medical Center, Paracelsus Medical University, Salzburg, Austria; ^2^Department of Psychiatry and Psychotherapy, Christian Doppler Medical Center, Paracelsus Medical University, Salzburg, Austria; ^3^Department of Critical Care, University College of London Hospital, London, United Kingdom; ^4^Department of Neurology, Klinikum rechts der Isar, Technische Universität München, München, Germany

**Keywords:** autoimmune encephalitis, humoral immunity, critical care, neurodegeneration, seizures, prognosis

## Abstract

**Background:**

Prevention and early recognition of critical illness in patients with autoimmune encephalitis (AE) is essential to achieve better outcome.

**Aim of the study:**

To evaluate risk factors for intensive care unit (ICU) admission and its prognostic impact in patients with AE.

**Patients and methods:**

A reclassification of patients hospitalized between 2011 and 2016 revealed 17 “definite” and 15 “probable” AE cases. Thirteen patients (41%) developed critical illness and required ICU admission. The underlying conditions were intractable seizures or status epilepticus (54%), altered mental state (39%), and respiratory failure (8%).

**Results:**

ICU admission was associated with longer time from first symptoms to hospitalization (*p* = 0.046). Regression analysis revealed that anemia on hospital admission and definite diagnosis of AE was associated with a higher risk of acquiring critical illness. At last follow-up after a median of 31 months (range 2.5–52.4), seven patients had died (23%) and 63% had a good outcome [modified Rankin Scale (mRS) 0–3]. Anemia was associated with poor prognosis (*p* = 0.021), whereas development of critical illness did not impact mortality and functional outcome.

**Conclusion:**

We confirmed the need for ICU care in a subgroup of patients and the prevailing objective is improved seizure control, and definite diagnosis of AE and anemia were identified as risk factors for development of critical illness. However, prognosis was not affected by ICU admission.

## Introduction

Autoimmune encephalitis (AE) refers to a group of heterogeneous immune-mediated disorders of the central nervous system. This emerging entity is characterized by the frequent detection of antibodies directed against proteins and/or receptors on the brain cell surface or intracellular antigens [such as Hu or Ma2, and less frequently collapsin response mediator protein 5 (CV2/CRMP5) and amphyphysin]. AE is assumed to comprise about 20% or more of all adult encephalitis cases (1). A further increase in its prevalence can be anticipated as new neuronal antibodies are discovered and updated diagnostic criteria implemented into clinical practice (2, 3). Good outcome at discharge was ascertained in 51% of patients with viral encephalitis, 41% with AE, and 54.2% with unknown or other etiologies. It must be assumed that the latter group also comprised additional AE cases (4).

The spectrum of clinical symptoms at presentation and acute course AE is wide, and there is clearly room for improvement regarding awareness, early recognition, and initiation of appropriate therapy (5, 6). Early recognition enables a more focused management of complications including conditions leading to critical illness and requiring intensive care unit (ICU) admission (7, 8). Patients with AE require ICU care for improved management of seizures, agitation, autonomic instability, and respiratory failure (7, 9, 10). Timely diagnosis and initiation of immunotherapy is a key prognostic factor (8, 11). Development of life-threatening complications requiring ICU admission has been linked to a prolonged hospital stay and lower likelihood of favorable outcome from AE (12, 13). Unraveling risk factors for the development of critical illness in patients with AE could help to improve morbidity and mortality of this condition by allowing for early monitoring and aggressive supportive care.

In this study, we aimed to unravel risk factors for development of critical illness in 32 patients with AE. Characteristics of 13 study patients have been reported in a previous study (7).

## Patients and Methods

### Study Design

This study was designed as a retrospective, electronic chart review of all adult patients admitted to the Department of Neurology at the Christian Doppler Medical Center between January 2011 and December 2016. This tertiary care center comprises 117 neurological beds including 9 ICU beds. The local Ethics Committee evaluated the study protocol (Ethikkommission für das Bundesland Salzburg; 415-EP/73/534-2015). No patient consent was required due to the non-interventional design according to national regulations.

### Patient Selection and Definition of AE

We screened the electronic hospital documentation system for potential patients by using variants and combinations of the following search terms: encephalitis, meningoencephalitis, autoimmune, immune-mediated, non-infectious, aseptic, central nervous system, unknown etiology, and seronegative. We included all patients who fulfilled the diagnostic criteria for either “definite” or “probable” AE as proposed by Mittal et al. (2) and Graus et al. (3).

Briefly, “definite” AE was diagnosed if an antibody against neuronal cell surfaces, synaptic or onconeuronal proteins was detected in the cerebrospinal fluid and/or serum. Tissue-based assay, cell-based immunoassay, and immunoblotting were used. We adhered to positivity thresholds suggested by the manufacturer or the specialized laboratory. The antibodies tested included Hu, Ma, Ri, Yo, Sox1, delta/notch-like epidermal growth factor-related receptor, CV2/CRMP5, glutamic acid decarboxylase, dipeptidyl-peptidase-like protein-6, metabotropic glutamate receptor 1, voltage-gated potassium channel-complex (VGKC) including leucine-rich glioma-inactivated 1 and contactin-associated protein-like 2 (CASPR2), *N*-methyl-d-aspartate (NMDA) receptor, γ-aminobutyric acid-B (GABA-B) receptor, α-amino-3-hydroxy-5-methyl-4-isoxazolepropionic acid (AMPA) receptor, and amphyphysin.

Cases classified as “probable” AE had to fulfill at least three of the subsequent criteria:

– classic phenotype with subacute onset,
– ≥1 antibody not meeting criteria for “definite” diagnosis (anti-neuronal or non anti-neuronal),
– inflammatory cerebrospinal fluid: two or more of the following: pleocytosis, elevated IgG synthesis rate, increased protein concentration, oligoclonal bands,
– ≥1 accompanying autoimmune disease, magnetic resonance imaging changes suggestive of encephalitis: mesial temporal or subcortical hyperintensity on fluid-attenuated inversion recovery/T2 imaging (3, 14),
– positive response to immunotherapy,
– detection of a neoplasm.

### Data Collection and Availability

Demographic data, past medical history, as well as the time between first symptoms and hospital admission, diagnosis and the first immunotherapy, underlying causes for the development of critical illness and subsequent ICU admission, length of ICU and hospital stay, and mortality were recorded. We further extracted clinical symptoms and the results of laboratory studies at hospital admission, as well as the most aberrant findings of magnetic resonance imaging and electroencephalography.

In patients admitted to the ICU, the Simplified Acute Physiology Score (SAPS) II, need for and duration of invasive mechanical ventilation, as well as the length of ICU stay were documented. Using structured telephone interviews, the modified Rankin Scale (mRS) was documented at a median of 31 (range 2.5–52.4) months after hospital admission in order to evaluate the degree of disability in all surviving patients. A favorable functional outcome was defined as a modified Rankin Scale (mRS) count of 0–3 points, whereas poor outcome was considered to be present if the modified Rankin Scale (mRS) exceeded 3 points (15). All data generated or analyzed during this study are included in this published article (and its Supplementary Material).

### Statistical Analysis

All statistical analyses were conducted using the IBM SPSS 23.0 software package (SPSS, Chicago, IL, USA). Descriptive methods were used to present data. Demographic, clinical, and paraclinical data were compared between groups using the Fisher’s exact or Mann–Whitney *U* test, as appropriate. Univariate and multivariate logistic regression analyses were used to identify independent risk factors associated with the need for ICU admission. Only variables with a *p* < 0.01 between critically and non-critically ill patients were included in the multivariate logistic regression analysis. All data are presented as median values with interquartile ranges, if not otherwise indicated. All reported *p*-values are two-tailed and considered to indicate statistical significance if <0.05.

## Results

**Figure 1:**
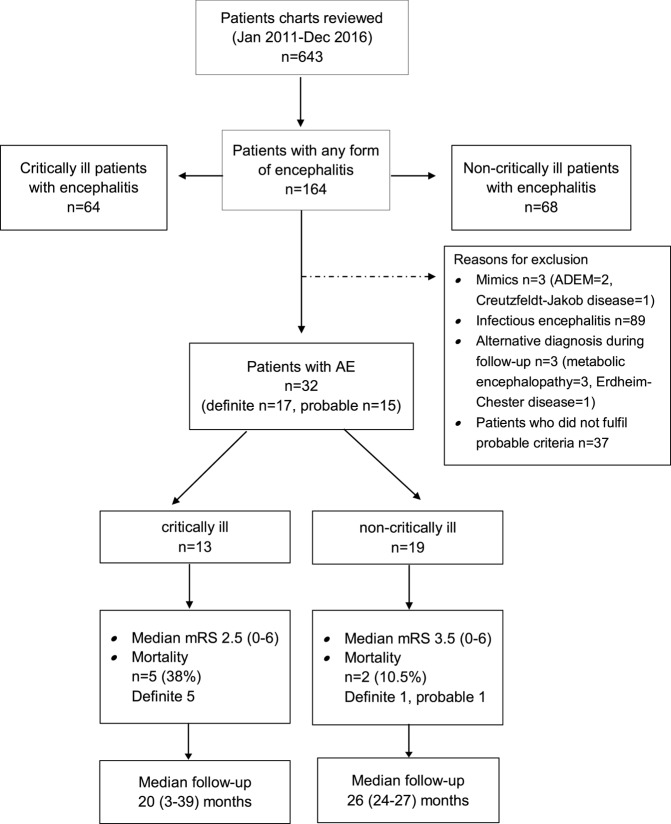
Patient selection and outcome.

The search of the electronic database resulted in 486 potential encephalitis cases. We identified 32 patients with AE (definite AE, *n* = 17; probable AE, *n* = 15) (Figure 1).

The following neuronal antibodies were identified in patients with definite AE: anti-NMDA (*n* = 3), VGKC (including LG1, CASPR) (*n* = 6), GABA-B (*n* = 2), Yo (*n* = 1), Hu (*n* = 1), AMPA (*n* = 2), Ma1/Ma2 (*n* = 1), and CV2/CRMP5 (*n* = 1). The antibodies detected in the probable AE cases were anti-Thyroperoxidase antibodies (TPOAb, *n* = 3), anti-Saccaromyces cerevisiae antibodies (ASCA, *n* = 1) and anti-Nuclear antibody (ANA, *n* = 1). A cerebrospinal fluid pleocytosis was found more frequently in patients with probable than definite disease (*p* = 0.02). Regarding immunotherapy, therapeutic plasma exchange was performed more commonly in patients with definite AE (*p* = 0.03). As shown in Tables S1 and S2 in Supplementary Material, we could not identify differences in other variables between patients with definite vs. probable AE.

Characteristics as well as therapeutic and diagnostic details of the study population are summarized in Tables 1 and 2.

**Table 1:** Clinical and demographic findings of 32 patients with autoimmune encephalitis.

	All patients	Critically ill	Non-critically ill	*p*-Value
*N*	32	13	19	
Age, years	64 (54–73)	64 (55–66)	66 (46–75)	0.4
Male gender (*n*/%)	22 (69)	11 (85)	11 (58)	0.14
**Comorbidities (*n*/%)**				
Arterial hypertension	15 (47)	6 (46)	9 (48)	1.0
Malignancy^a^	10 (31)	4 (31)	6 (32)	1
Hyperlipidemia	9 (28)	6 (46)	3 (16)	0.1
Nicotine abuse^b^ (*n*/%)	9 (31)	4 (31)	5 (31)	1
Autoimmune disease^c^	7 (22)	2 (15)	5 (26)	1
Type 2 diabetes mellitus	6 (19)	4 (31)	2 (11)	0.19
Hypothyroidism	4 (13)	3 (23)	1 (5)	0.28
Alcohol abuse^b^ (*n*/%)	3 (10)	1 (8)	2 (13)	1
Charlson’s comorbidity index	2 (1–4)	2 (2–5)	2 (1–3)	0.23
**Presenting symptoms (*n*/%)**				
Altered mental state^d^	21 (66)	7 (54)	14 (74)	0.28
Seizures	14 (44)	6 (46)	8 (42)	1
Memory loss	9 (28)	4 (31)	5 (26)	1
Movement disorder	5 (16)	1 (8)	4 (21)	0.63
Headache	5 (16)	2 (15)	3 (16)	1
Speech impairment	2 (6)	0 (0)	2 (11)	0.5
Time between first symptoms and hospitalization (days)	14 (4–96)	91 (0–180)	10 (1–30)	**0.046**
Time between the hospital admission and diagnosis (days)	5.5 (3–35)	3 (3–6)	16 (3–59)	**0.04**
Hospital length of stay (days)	12.5 (9–22.25)	15 (10–37)	11 (7–21)	**0.03**

*Data are given as median values with interquartile range, unless otherwise specified*.

*Values are in bold if they were p<0.05 or p<0.01*.

*^a^Small cell lung cancer (*n* = 3), ovarian adenocarcinoma (*n* = 1), ovarian teratoma (*n* = 1), prostate cancer (*n* = 1), pancreatic cancer (*n* = 1), testicular cancer (*n* = 1), colorectal cancer (*n* = 1), and lymphoma (*n* = 1)*.

*^b^Information available in 29 patients*.

*^c^Hashimoto’s thyreoiditis (*n* = 3), psoriasis (*n* = 2), Crohn’s disease (*n* = 1), and vitiligo (*n* = 1)*.

*^d^Defined as having one or more of the following symptoms: confusion, not acting right, altered behavior, generalized weakness, lethargy, agitation, psychosis, disorientation, inappropriate behavior, inattention, and hallucinations*.

**Table 2:** Diagnostic findings and therapies of 32 patients with autoimmune encephalitis.

	All patients	Critically ill	Non-critically ill	*p*-Value
*N*	32	13	19	
Definite diagnosis of AE (*n*/%)	17 (53)	10 (77)	7 (37)	**0.04**
Time between first symptoms and diagnosis (days)	15 (5–30)	28 (11–110.5)	13.5 (0.25–27.25)	0.14
Laboratory findings				
Anemia on hospital admission^a^	15 (47)	10 (77)	5 (26)	**0.01**
Low plasma protein levels^a^	14 (45)	6 (46)	8 (44)	1
Low white blood cell count^a^	8 (25)	3 (23)	5 (26.3)	1
Elevated gamma-GT serum levels	7 (23)	5 (39)	2 (12)	0.19
Elevated vitamin B12 levels^a^	8 (27)	4 (33)	4 (22)	0.67
Elevated folic acid levels^a^	11 (41)	3 (27)	8 (50)	0.42
Abnormal MRI findings^b^ (*n*/%)	21 (70)	8 (62)	13 (77)	0.44
Abnormal EEG findings^b^ (*n*/%)	26 (87)	11 (85)	15 (88)	1
Inflammatory CSF^b^ (*n*/%)	18 (58)	8 (62)	10 (56)	1
Pleocytosis^c^	19 (61)	9 (69)	10 (53)	0.48
Elevated IgG synthesis^c^	14 (45)	8 (62)	6 (33)	0.15
Oligoclonal bands^c^	3 (9)	2 (15)	1 (5)	0.55
Time between onset of symptoms and first immunotherapy (days)	30 (8–94)	81 (14–157)	25.5 (8–57)	0.24
Time between hospital admission and first immunotherapy (days)	16 (5–56)	8 (3–22)	17 (5–59)	0.19
Immunotherapy (*n*/%)	26 (81)	11 (42)	15 (58)	1
Corticosteroids	16 (50)	9 (69)	7 (37)	0.14
Intravenous IgG	19 (59)	7 (54)	12 (63)	0.72
Therapeutic plasma exchange	11 (34)	6 (46)	5 (26)	0.28
Rituximab	2 (6)	1 (8)	1 (5)	1
Cyclophosphamide	1 (3)	0 (0)	1 (5)	1
Improvement after first line immunotherapy (*n*/%)	21 (66)	7 (54)	14 (74)	0.28
Improvement after second line immunotherapy (*n*/%)	1 (3)	0 (0)	1 (5)	1

*MRI, magnetic resonance imaging; EEG, electroencephalogram; CSF, cerebrospinal fluid*.

*Values are in bold if they were p<0.05 or p<0.01*.

*Data are given as median values with interquartile range, unless otherwise specified*.

*^a^Normal values: anemia was defined <12.0 g/dl (female) and 13.5 g/dl (male). Hypoproteinemia < 6.6 mg/dl, Leukocytes < 4.3 G/l, gamma-GT < 71.0 U/l, vitamin B12 < 663 pg/ml, folic acid < 9.1 ng/ml*.

*^b^Availability of data: 30 had data of MRI and EEG, n = 31 CSF, n = 30 vitamin B12 level, n = 27 folic acid level, n = 31 protein serum level, n = 30 gamma-GT serum level*.

*^c^pleocytosis: > 4 cells/μl. Normal values for IgG: <5.86 mg/dl*.

Altered mental state (*n* = 5), seizures (*n* = 4), status epilepticus (*n* = 3), and tetraparesis leading to respiratory insufficiency were reasons for ICU admission in 17 study patients. Two of these patients required endotracheal intubation and invasive mechanical ventilation due to respiratory failure (*n* = 1) and status epilepticus (*n* = 1). Percutaneous tracheotomy was performed in one patient. AE patients who developed critical illness experienced a longer time delay between the development of first symptoms and hospital admission as well as between hospital admission and diagnosis than non-critically ill patients. Definite diagnosis of AE was more common and anemia (at hospital admission) was more frequently detected and the duration of hospital stay was longer in critically than non-critically ill study patients. None of the patients had thrombocytopenia.

In the univariate regression analysis, definite diagnosis of AE and anemia on admission predicted risk for ICU admission (Table 3).

**Table 3:** Uni- and multivariate analysis of risk factors for intensive care unit admission in patients with autoimmune encephalitis.

Variables		Univariate analysis		Multivariate analysis		

	OR	95% CI	*p-*Value	OR	95% CI	*p-*Value
Definite diagnosis of AE	5.7	1.163–28.069	**0.032**	6.6	0.789–54.635	**0.082**
Anemia on hospital admission	9.3	1.801–48.375	**0.008**	8.8	1.216–63.646	**0.031**
Altered mental state as a reason for hospital admission	4.5	0.986–20.354	0.052	2.5	0.345–17.932	0.366
Hyperlipidemia as comorbidity	4.6	0.881–23.710	0.070	5.3	0.547–50.627	0.15

*OR, odds ratio; 95% CI, 95% confidence interval*.

*Values are in bold if they were p<0.05 or p<0.01*.

We found a trend for altered mental status as reason for hospital admission and hyperlipidemia as additional risk factors. The multivariate analysis disclosed anemia at hospital admission as an independent risk factor for the development of life-threatening complications and ICU admission, whereas the definite diagnosis showed a trend (Table 3).

Functional outcome was determined at a median of 31 (range 2.5–52.4) months after hospital admission (Figure 1). Two study patients were lost to follow-up as they could not be contacted by telephone. Seven patients (23%) died during this observation period (critically ill patients, *n* = 5; non-critically ill patients, *n* = 2). Details about the causes of death were available in five patients. Causes of death were tumor progression (*n* = 3), cardiorespiratory failure, and complications of heart surgery (*n* = 1 each). Favorable functional outcome [modified Rankin Scale (mRS) 0–3] was recorded in 19 patients (63.3%) (6/13, 31.6%) and non-critically ill AE patients (6/19, 72%; *p* = 0.27). Except for a higher rate of anemia at hospital admission among study patients with poor functional outcome, we did not identify any differences in demographic data, past medical history, immunotherapies, and time to treatments or cerebrospinal fluid profiles between patients with favorable and poor functional outcome (Tables S3 and S4 in Supplementary Material).

Patient outcome was determined at a median of 31 months (range 2.5–52.4) from hospital admission. Seven patients had died (23%), among were five of the ICU and two from the non-ICU cohort. The median modified Rankin Scale (mRS) was 3 (range 0–6). Mortality and functional outcome did not differ between patients who did or did not develop critical illness (Figure 1). We did not identify differences with regard to comorbidities, immunotherapies, and time to these treatments, CSF profile and demographics except the higher rate of anemia among patients with poor outcome (*p* = 0.021), as shown in Table S2 in Supplementary Material.

## Discussion

In this retrospective chart review, we studied 32 patients with AE treated at a tertiary care hospital over the period of 2011–2016 and aimed to identify risk factors for the development of life-threatening conditions requiring ICU admission. The high disease burden related to AE was reflected by the need for ICU care in almost half of the subjects in this cohort, as well as a case fatality rate of 23% and poor functional long-term outcome in 37% of study patients. We identified definite AE, which is linked to the detection of an antibody against neuronal cell surfaces, synaptic or onconeuronal proteins, and the presence of anemia at hospital admission as independent risk factors for the development of critical illness. Intractable seizures and altered mental state were the predominant life-threatening conditions leading to ICU admission in our patients. The need for ICU care *per se* was not related to worse long-term outcome compared to patients who did not require ICU admission.

The significance of the identified risk factors for ICU admission in our cohort with 17 definite and 15 probable cases of AE needs to be entertained in detail. In the present study, the proportion of patients with definite diagnosis of AE (44%) was in the range of cohorts reported in the literature (7, 16, 17). In this context, we need to consider that the detection of an antibody might have raised the physician awareness for poor prognosis and ICU admission. In turn, diagnosis of seronegative cases can be cumbersome in clinical practice and may only be taken into account at a later time point of the disease. Our analysis, however, indicates that patients with definite disease seem to have had indeed more frequently conditions requiring ICU admission. A particularly proactive ICU admission would have been reflected by lower SAPS II scores in patients with definite AE. However, this was not observed in our cohort. Interestingly, the overall rate of endotracheal intubation and mechanical ventilation in patients admitted to the ICU was low (6.3%). The lower frequency of therapeutic plasma exchange in probable patients might be related to the assumption of lesser efficacy without detection of an antibody. In this regard, the study provides insights to the spectrum of probable AE cases defined by the new diagnostic criteria and importantly disclosed similar outcomes when compared with definite AE.

Anemia is highly prevalent in critically ill patients but the context in patients with AE remains unclear (18). A multitude of studies found that up to two-thirds of critically ill patients have a hemoglobin concentration <12 g/dl at ICU admission (19, 20). Irrespective of the underlying condition, a reduced hemoglobin concentration limits maximum systemic oxygen delivery and therefore makes patients more likely to develop life-threatening conditions and require intensive care. The consequences of anemia may be aggravated by altered mental status and seizures, which *per se* can also cause insufficient oxygen supply to the brain (21, 22).

The most common initial symptoms and reasons for hospital and ICU admission were altered mental state and seizures, which is consistent with the literature (16, 17, 23–25). Of note, four patients presented with status epilepticus to the emergency ward. We could not identify specific signs and symptoms, laboratory and imaging findings or demographic features, which were associated with a higher risk of critical illness. In our study, critically ill patients had a longer time between first symptoms and hospitalization and an overall longer hospital stay. Furthermore, comorbidities did not affect the need for ICU admission. This contrasts our previous findings of increased probability of death among critically ill AE patients with higher Charlson’s comorbidity index (7). Potential reasons may be the more historical period (2002–2015) with limited recognition and knowledge of the condition. In addition, a more aggressive treatment approach and prevention of detrimental courses by proactive ICU admission in more recent years might have played a role. Moreover, we could not confirm the observations from mostly larger but historical patient series which disclosed an association of age, ICU admission, and poor functional outcome (4, 8, 13, 26). Thus, whether increased earlier diagnosis, improved understanding, and an altered treatment approach are responsible for our observation, needs to be confirmed in future independent studies. Early initiation of immunotherapy can effectively improve outcome from AE. Yet, fulminant cases remain a challenge and fatal cases are still seen (27). The relatively high rate of ICU admissions in our cohort may therefore reflect both the frequent occurrence of and the increasing knowledge about potentially critical conditions and intention to prevent detrimental courses in patients with AE. Yet, it needs to be acknowledged that there is only limited knowledge about specific ICU care in patients with AE beyond aggressive immunosuppression and therapeutic plasma exchange (8, 15, 16, 26). Unlike in other neurologic conditions requiring ICU admission, the SAPS II was not predictive of neurological outcome in our cohort.

Our study has some limitations beyond the restricted cohort size. First, a retrospective single center study with local customs for ICU referrals and non-standardized approach toward antibody testing in potential encephalitis cases is likely to interfere with the generalizability of the findings. Secondly, awareness for seronegative AE increased just recently and this group of patients could be therefore under-represented in our study. This supports the need for future inception studies to clarify the sensitivity and specificity of the diagnostic criteria for probable disease.

In conclusion, definite diagnosis and the presence of anemia at hospital admission were independent risk factors for the development of life-threatening conditions requiring ICU admission in 32 patients with AE. Strikingly, the need for ICU care was not related to worse functional long-term outcome in this cohort.

## Ethics Statement

Ethikkommission für das Bundesland Salzburg; 415-EP/73/534-2015.

## Author Contributions

GH, LH, MD, and JS conceived and designed the study. GH, LH, and JS searched and evaluated the cases. GH, LH, MD, TM, SP, ML, HN, ET, and WA analyzed the data. GH, LH, MD, and JS wrote the paper and prepared all figures and supplements. All authors reviewed and edited the manuscript. All authors read and approved the final version of the manuscript.

## Conflict of Interest Statement

The authors declare that the research was conducted in the absence of any commercial or financial relationships that could be construed as a potential conflict of interest.
