# Supplementary material for: Risk Factors for Intensive Care Unit Admission in Patients with Autoimmune Encephalitis
Source: Front Immunol. 2017 Jul 28;8:835. doi: 10.3389/fimmu.2017.00835 (PMC5532517; doi:10.3389/fimmu.2017.00835)
Supplement: Supplementary file 1 [file Table_1.DOCX]

Table S1. Outcome of patients with AE, part 1.

|  | All patients | Favourable outcome | Poor outcome | *p* value |
| --- | --- | --- | --- | --- |
| n | 30* | 19 | 11 |  |
| Age (years) | 64 (51-73) | 57 (46-73) | 64 (61-84) | 0.18 |
| Male gender (n/%) | 22 (73) | 15 (79) | 7 (63) | 0.42 |
| Comorbidities (n/%) |  |  |  |  |
| Arterial hypertension | 15 (50) | 8 (42) | 7 (63) | 0.45 |
| Malignancy | 10 (33.3) | 5 (26) | 5 (45) | 0.43 |
| Hyperlipidaemia | 8 (26.6) | 4 (21) | 4 (36) | 0.42 |
| Nicotine abuse** (n/%) | 8 (26.6) | 4 (23) | 4 (40) | 0.415 |
| Autoimmune disease | 7 (23.3) | 4 (21) | 3 (27) | 1.0 |
| Type 2 diabetes mellitus | 5 (16.6) | 2 (10.5) | 3 (27) | 0.33 |
| Hypothyroidism | 3 (10) | 1 (5) | 2 (18) | 0.54 |
| Alcohol abuse** (n/%) | 3 (10) | 2 (11) | 1 (10) | 1 |
| Charlson’s Comorbidity Index | 2 (1-4) | 5 () | 3 (3) | 0.054 |
| Time between first symptoms and hospitalization (days) | 14 (4-96) | 10 (10-95) | 21 (7-96) | 0.4 |
| Presenting symptoms (n/%) |  |  |  |  |
| Altered mental stare | 20 (67) | 11 (58) | 9 (81) | 0.25 |
| Seizures | 12 (40) | 8 (42) | 4 (36) | 1 |
| Memory loss | 8 (27) | 4 (21) | 4 (36) | 0.42 |
| Movement disorder | 5 (17) | 2 (11) | 3 (27) | 0.33 |
| Headache | 5 (17) | 4 (21) | 1 (9) | 0.63 |
| Speech impairment | 2 (6.6) | 0 (0) | 2 (18) | 0.13 |
| Hospital length of stay (days) | 14 (9-22) | 11 (10-21) | 19 (11-42) | 0.18 |
| Detection of neuronal antibody, n % | 15 (50) | 8 (42) | 7 (63) | 0.45 |
| Need for ICU, n % | 12 (40) | 6 (32) | 6 (55) | 0.27 |
| Data shown as median (interquartile range) unless otherwise specified  Fisher exact test and Mann-Whitney test were used to perform this comparison.  * 2 patients were not available by telephone  ** Data were available for 27 patients (good outcome n=17, poor outcome n=10) | | | | |
