# Supplementary material for: Risk Factors for Intensive Care Unit Admission in Patients with Autoimmune Encephalitis
Source: Front Immunol. 2017 Jul 28;8:835. doi: 10.3389/fimmu.2017.00835 (PMC5532517; doi:10.3389/fimmu.2017.00835)
Supplement: Supplementary file 2 [file Table_2.DOCX]

**Table S2.** Outcome of patients with AE, part 2.

|  | All Patients | Favourable  outcome | Poor  outcome | *p*-value |
| --- | --- | --- | --- | --- |
| n | 30* | 19 | 11 |  |
| Detection of neuronal antibody (n/%) | 15 (50) | 8 (50) | 7 (50) | 0.45 |
| Time between first symptoms and diagnosis (days) | 15 (5-30) | 17 (0.25-74) | 15 (11-28) | 0.4 |
| Laboratory findings (n/%) |  |  |  |  |
| Anaemia† | 15 (50) | 6 (31.6) | 9 (82) | **0.02** |
| Plasma hypoproteinaemia | 14 (47) | 9 (47) | 5 (45) | 1 |
| Leucocytosis | 7 (23.3) | 3 (16) | 4 (36) | 0.37 |
| Elevated Gamma-GT serum levels# | 7 (25) | 2 (12) | 5 (45) | 0.08 |
| Abnormal MRI findings# (n/%) | 21 (75) | 13 (77) | 8 (73) | 1 |
| Abnormal EEG findings# (n/%) | 24 (86) | 14 (82) | 10 (91) | 1 |
| Inflammatory CSF# (n/%) | 17 (59) | 10 (55) | 7 (64) | 0.72 |
| Red blood cells detected | 20 (69) | 12 (75) | 8 (57) | 1 |
| Pleocytosis | 19 (66) | 12 (67) | 8 (73) | 0.69 |
| Elevated IgG synthesis | 13 (45) | 6 (33) | 7 (63) | 0.14 |
| Oligoclonal bands | 3 (10) | 3 (16) | 0 | 0.28 |
| Elevated vitamin B12 levels | 8 (28) | 3 (16) | 5 (50) | 0.08 |
| Elevated folic acid levels | 11 (42) | 6 (33) | 5 (62) | 0.22 |
| Time between onset of symptoms and first immunotherapy (days, range) | 30 (8-94) | 26 (6-113) | 44 (20-81) | 0.32 |
| Time between hospital admission and first immunotherapy (days, range) | 16 (5-56) | 11.5 (5-59) | 19 (5-28) | 0.66 |
| Immunotherapy (n/%) | 24 (80) | 17 (89.5) | 10 (64) | 0.16 |
| Corticosteroids | 15 (50) | 8 (42) | 7 (64) | 0.45 |
| Intravenous IgGs | 17 (57) | 12 (63) | 5 (46) | 0.45 |
| Therapeutic plasma exchange | 11 (37) | 7 (37) | 4 (36) | 1 |
| Rituximab | 2 (7) | 0 (0) | 2 (18) | 0.13 |
| Cyclophosphamide | 1 (3.3) | 0 (0) | 1 (9) | 0.37 |
| Improvement after 1^st^ line immunotherapy (n/%) | 19 (63) | 14 (74) | 5 (46) | 0.24 |
| Improvement after 2^nd^ line immunotherapy (n/%) | 1 (3.3) | 0 (0) | 1 (9) | 0.37 |
| MRI, magnetic resonance imaging; EEG, electroencephalogram; CSF, cerebrospinal fluid.  Data are given as median values with interquartile range, unless otherwise specified.  * 2 patients could not be contacted  # missing data; 28 patients had data of MRI and EEG, n=29 CSF finding, n=26 folic acid level, n=30 oligoclonal bands, n=28 Gamma-GT serum level  † Anaemia was defined less than 12.0 g/dl (female) and 13.5 g/dl (male) | | | | |
