# Supplementary material for: Risk Factors for Intensive Care Unit Admission in Patients with Autoimmune Encephalitis
Source: Front Immunol. 2017 Jul 28;8:835. doi: 10.3389/fimmu.2017.00835 (PMC5532517; doi:10.3389/fimmu.2017.00835)
Supplement: Supplementary file 3 [file Table_3.DOCX]

**Table S3.** Cohort of 32 patients with AE: definite and probable diagnosis, part 1.

|  | All  patients | Definite diagnosis | Probable  diagnosis | *p* value |
| --- | --- | --- | --- | --- |
| n | 32 | 17 | 15 |  |
| Age (years) | 64 (54-73) | 64 (49-73) | 61 (46-74) | 0.88 |
| Male gender (n/%) | 22 (69) | 11(65) | 11 (73) | 0.71 |
| Comorbidities (n/%) |  |  |  |  |
| Arterial hypertension | 15 (47) | 7 (41) | 8 (53) | 0.72 |
| Malignancy | 10 (31.3) | 7 (41) | 3 (20) | 0.27 |
| Hyperlipidaemia | 9 (28) | 5 (29) | 4 (27) | 1 |
| Nicotine abuse# | 9 (31) | 5 (31) | 4 (31) | 1 |
| Autoimmune disease | 7 (22) | 2 (12) | 5 (22) | 0.2 |
| Type 2 diabetes mellitus | 6 (19) | 3 (18) | 3 (20) | 1 |
| Hypothyroidism | 4 (12.5) | 2 (12) | 2 (13) | 1 |
| Alcohol abuse# | 3 (10) | 2 (13) | 1 (8) | 1 |
| Charlson’s Comorbidity Index | 2 (1-4) | 2.5 (2-5) | 2 (1-3) | 0.13 |
| Time between first symptoms and hospitalization (days) | 14 (4-96) | 29 (2-180) | 10 (5-30) | 0.55 |
| Initial symptoms (n/%) |  |  |  |  |
| Altered mental state | 21 (65) | 11(65) | 10 (67) | 1 |
| Seizures | 14 (44) | 8 (47) | 6 (40) | 0.74 |
| Memory loss | 9 (28) | 5 (29) | 4 (27) | 1 |
| Movement disorder | 5 (16) | 3 (18) | 2 (13) | 1 |
| Headache | 5 (16) | 1 (6) | 4 (27) | 0.16 |
| Speech impairment | 2 (6.3) | 1 (6) | 1 (7) | 1 |
| Reasons for hospital admission (n/%) |  |  |  |  |
| Altered mental state | 13 (40) | 10 (59) | 3 (20) | **0.036** |
| Seizure | 11 (34) | 6 (35) | 5 (33) | 1.0 |
| Status epilepticus | 4 (12.5) | 1 (6) | 3 (20) | 0.32 |
| Flu symptoms | 1 (3) | 0 (0) | 1 (7) | 0.47 |
| Unsteady gait | 1 (3) | 0 (0) | 1 (7) | 0.47 |
| Dizziness | 1 (3) | 0 (0) | 1 (7) | 0.47 |
| Ataxia | 1 (3) | 0 (0) | 1 (7) | 0.47 |
| SAPS II (points) | 21(13-31) | 22.5 (17-31) | 13 (13-0) | 0.63 |
| Hospital length of stay (days) | 12.5 (9-22) | 15 (7-29) | 11 (10-22) | 0.6 |
| SAPS II, Simplified Acute Physiology Score II.  Data shown as median (interquartile range) unless otherwise specified  Fisher exact test and Mann-Whitney test were used to perform this comparison.  # missing data: 29 patients had data of alcohol and nicotine abuse | | | | |
