# Supplementary material for: Risk Factors for Intensive Care Unit Admission in Patients with Autoimmune Encephalitis
Source: Front Immunol. 2017 Jul 28;8:835. doi: 10.3389/fimmu.2017.00835 (PMC5532517; doi:10.3389/fimmu.2017.00835)
Supplement: Supplementary file 4 [file Table_4.DOCX]

**Table S4.** Cohort of 32 patients with AE: definite and probable diagnosis, part 2.

|  | All Patients | Definite  diagnosis¶ | Probable diagnosis† | *p*-value |
| --- | --- | --- | --- | --- |
| n | 32 | 17 | 15 |  |
| Time between first symptoms and diagnosis (days) | 15 (5-30) | 19.5 (0-34) | 15 (6-60) | 0.94 |
| Laboratory findings (n/%) |  |  |  |  |
| Anaemia† | 15 (47) | 9 (53) | 6 (40) | 0.5 |
| Plasma hypoproteinaemia# | 14 (45) | 5 (31) | 9 (60) | 0.16 |
| Leucocytosis | 8 (25) | 4 (23) | 4 (27) | 1.0 |
| Elevated Gamma-GT serum levels# | 7 (23) | 6 (37) | 1 (7) | 0.09 |
| Abnormal MRI findings# (n/%) | 21 (70) | 11 (69) | 10 (71) | 1 |
| Abnormal EEG findings# (n/%) | 26 (87) | 13 (81) | 13 (93) | 0.6 |
| Inflammatory CSF# (n/%) | 18 (58) | 9 (60) | 9 (56) | 1 |
| Red blood cells detected | 22 (71) | 12 (75) | 10 (67) | 0.7 |
| Pleocytosis | 19 (61) | 7 (44) | 13 (87) | **0.02** |
| Elevated IgG synthesis | 14 (45) | 8 (50) | 6 (40) | 0.72 |
| Oligoclonal bands | 3 (9) | 1 (6) | 2 (13) | 0.59 |
| Elevated vitamin B12 levels | 8 (27) | 5 (33) | 3 (20) | 0.68 |
| Elevated folic acid levels | 11 (41) | 6 (46) | 5 (37) | 0.7 |
| Time between onset of symptoms and first immunotherapy (days) | 30 (8-94) | 37 (9-91) | 28 (8-112) | 0.78 |
| Time between hospital admission and first immunotherapy (days) | 16 (5-56) | 42 (11-80) | 8 (4-19) | 0.1 |
| Immunotherapy (n/%) | 26 (81) | 14 (82) | 12 (80) | 1 |
| Corticosteroids | 16 (50) | 7 (41) | 9 (60) | 0.48 |
| Intravenous IgGs | 19 (59) | 10 (59) | 9 (60) | 1 |
| Therapeutic plasma exchange | 11 (34) | 9 (53) | 2 (13) | **0.03** |
| Rituximab | 2 (6) | 1 (6) | 1 (7) | 1.0 |
| Cyclophosphamide | 1 (3) | 0 (0) | 1 (7) | 0.47 |
| Improvement after 1^st^ line immunotherapy (n/%) | 21 (66) | 9 (53) | 12 (80) | 0.15 |
| Improvement after 2^nd^ line immunotherapy (n/%) | 1 (3) | 0 (0) | 1 (7) | 0.47 |
| Favourable outcome* (mRS 0-3) | 19 (63) | 8 (53) | 11 (73) | 0.45 |
| MRI, magnetic resonance imaging; EEG, electroencephalogram; CSF, cerebrospinal fluid, mRS, modified Rankin Scale,  Data are given as median values with interquartile range, unless otherwise specified.  * 2 patients were not available by telephone (definite, n=2).  # missing data: 30 patients had data of MRI and EEG, n=31 CSF, n=30 Vitamin B12, n=27 folic acid level, n=31 serum protein level, n=30 Gamma-GT serum level  † Anaemia was defined less than 12.0g/dl (female) and 13.5g/dl (male) | | | | |
